# Higher tendency of undertreatment in older patients with laryngeal cancer in Brazil

**DOI:** 10.1590/1980-549720250031

**Published:** 2025-06-02

**Authors:** Ana Luiza Fassizoli da Fonte, Guilherme Jorge Costa, Adilis Stepple da Fonte, Rodrigo Alves Pinto, Maria Júlia Gonçalves de Mello

**Affiliations:** I Instituto de Medicina Integral Professor Fernando Figueira, Department of Radiotherapy – Recife (PE), Brazil.; II Hospital de Câncer de Pernambuco, Department of Education and Research – Recife (PE), Brazil.; III Instituto de Medicina Integral Professor Fernando Figueira, Department of Pneumology – Recife (PE), Brazil.; IV Hospital de Câncer de Pernambuco, Department of Head and Neck Surgery – Recife (PE), Brazil.; V Instituto de Medicina Integral Professor Fernando Figueira, Department of Oncology – Recife (PE), Brazil.; VI Instituto de Medicina Integral Professor Fernando Figueira, Clinical Research Division – Recife (PE), Brazil.

**Keywords:** Undertreatment, Neoplasm staging, Laryngeal neoplasms, Aged, Brazil, Subtratamento, Estadiamento de neoplasias, Neoplasias laríngeas, Idoso, Brasil

## Abstract

**Objective:**

This study compared treatments and outcomes between patients younger than 70 years and those aged 70 years or older (elderly) with laryngeal cancer (LC).

**Methods:**

Data were collected from Brazilian hospital records between 2000 and 2017.

**Results:**

A total of 38,978 patients were analysed, of whom 8,803 (22.6%) were ≥70 years old. Elderly patients were more frequently diagnosed at early stages (39.3% vs. 28.9%; p<0.001). However, these patients were 15% less likely to undergo surgery, 46% less likely to receive chemotherapy, and 33% less likely to receive combined treatments. In addition, elderly patients were 35% more likely to receive supportive care only.

**Conclusions:**

Despite early diagnosis, elderly patients received fewer oncological treatments and more palliative care.

## RESUMO

**Objetivo:**

Este estudo comparou tratamentos e resultados em pacientes com câncer de laringe (LC) com menos de 70 anos e 70 anos ou mais (idosos).

**Métodos:**

Os dados foram coletados de registros hospitalares brasileiros entre 2000 e 2017.

**Resultados:**

No total, foram analisados 38.978 pacientes, sendo 8.803 (22,6%) idosos. Entre os idosos, houve maior frequência de diagnósticos em estágios iniciais (39,3 contra 28,9%; p<0,001), no entanto esses pacientes foram 15% menos propensos a realizar cirurgia, 46% menos a receber quimioterapia e 33% menos a receber tratamentos combinados. Além disso, apresentaram 35% mais chance de receber apenas cuidados de suporte.

**Conclusões:**

Apesar do diagnóstico precoce, os pacientes idosos receberam menos tratamentos oncológicos e mais cuidados paliativos.

## INTRODUCTION

Globally, 188,960 new laryngeal cancer (LC) cases were predicted for 2022^1^ and LC remains the second most common head and neck cancer. In Brazil, around 7,790 new cases of LC are expected between 2023 and 2025^2^. Brazilian LC patients are predominantly men (ratio 6.5/1), with an average age of 61 years, and 23.2% were over 70 years old^3^. Older patients with cancer used to be undertreated, overtreated, or excluded from clinical trials^4^.

The TNM (Tumor, Node, Metastasis) system classifies LC into early (I and II), locally advanced (III, IVa, and IVb), and metastatic stages (IVc). Surgery or exclusive radiotherapy is recommended for early LC. In locally advanced cases, chemotherapy with radiotherapy can preserve the larynx, offering overall survival comparable to surgery followed by radiotherapy. Extensive lesions (T4) or poor pre-treatment function may require total laryngectomy followed by radiotherapy, with or without chemotherapy^5^.

Disparities in cancer treatment by age are less well-defined in patients with LC, particularly among those aged ≥70 years. Previous findings have highlighted distinct treatment patterns and outcomes in this group^6^, aligning with international studies that commonly use this age cutoff for comparative oncology research. Therefore, this study aims to compare cancer treatment modalities and responses between patients aged <70 years and those ≥70 years.

## METHODS

This ecological study used data from patients with LC (C32) between 2000 and 2017 obtained from hospital-based registries using the Brazilian National Cancer Institute (INCA) integrated database, involving 287 hospitals in 26 states and the Federal District. Data were obtained on December 5, 2019, at www.inca.gov.br. Individuals under 18 years of age or with incomplete data staging were excluded. We included LC cases histologically classified as non-squamous and squamous cell carcinoma.

The following variables were analyzed: age, sex, smoking, time of treatment initiation, LC stage (early, locally advanced, or metastatic), histology (squamous cell or non-squamous cell carcinoma), early death (at the end of the initial treatment; yes or survival), response to the initial treatment, and oncological treatment (support care, surgery, radiotherapy, or chemotherapy).

Ethical approval was not required as secondary data was used.

A descriptive analysis was conducted using means and standard deviations for the continuous variables and the distribution of frequencies for the categorical variables. The χ^2^ test was used to compare the frequency of the categorical variables. The odd ratios (OR) with 95% confidence intervals (95% CIs) were calculated initially using a univariate analysis. These results were then adjusted according to sex, smoking, and stage using a multiple logistic regression model. The forward stepwise selection method was used in the regression analysis. Differences were considered statistically significant when p<0.05.

## RESULTS

Data from 221,566 head and neck cancer patients revealed 57,727 (27%) had LC. After exclusions, 38,978 patients were included; 8,803 (22.6%) were older, and 33,885 (86.9%) were male. Regarding staging, 31.2, 58.9, and 2.2% had early, locally advanced, and metastatic stages, respectively. Radiotherapy (73.2%), chemotherapy (42.3%), surgery (40.4%), and support care (9.3%) were the most common treatments (Table 1).

**Table 1.** Demographic and clinicopathologic characteristics, and oncological modalities treatment, according to the staging of laryngeal cancer patients, Brazil, 2000–2017.

Characteristics	All patients n (%) 38,978 (100)	Young n (%) 30,175 (77.4)	Older n (%) 8,803 (22.6)	p-value*
Mean of age±SD (years)	61.2±10.7	56.9±7.8	75.8±5	<0.001
Median years (QII)	61	58	75	
Sex				
Male	33,885 (86.9)	26,240 (87)	7,645 (86.9)	0.788
Female	5,090 (13.1)	3,933 (13)	1,157 (13.1)	
Smoking				
Never	2,667 (13.7)	1,852 (12.4)	815 (18.1)	<0.001
Former smokers	3,587 (18.4)	2,541 (17)	1,046 (23.3)	
Smokers	13,193 (67.8)	10,558 (70.6)	2,635 (58.6)	
Time to initial treatment				
<60 days	20,029 (58.9)	15,510 (58.5)	4,519 (60.2)	0.008
≥60 days	13,988 (41.1)	11,001 (41.5)	2,987 (39.8)	
Staging				
Early	12,175 (31.2)	8,715 (28.9)	3,460 (39.3)	<0.001
Locally advanced	25,931 (58.9)	20,744 (68.7)	5,187 (58.9)	
Metastatic	872 (2.2)	716 (2.4)	156 (1.8)	
Oncological treatment (in general)				
Support care	3,634 (9.3)	2,635 (8.8)	999 (11.4)	<0.001
Surgery	14,218 (40.4)	11,403 (41.5)	2,815 (36.1)	<0.001
Radiotherapy	25,778 (73.2)	19,920 (72.6)	5,858 (75.2)	<0.001
Chemotherapy	14,895 (42.3)	12,574 (45.8)	2,321 (29.8)	<0.001
Oncological treatment at early staging				
Support care	1,004 (8.3)	686 (7.9)	318 (9.2)	0.018
Surgery	4,613 (41.4)	3,489 (43.6)	1,124 (35.8)	<0.001
Radiotherapy	7,961 (71.5)	5,617 (70.2)	2,344 (74.7)	<0.001
Chemotherapy	1,880 (16.9)	1,494 (18.7)	386 (12.3)	0.001
Response	4,096 (87.9)	2,989 (88.6)	1,107 (85.9)	0.014
Death	283 (5.2)	186 (4.8)	97 (6.3)	0.02
Oncological treatment at locally advanced stage				
Support care	2,498 (9.7)	1,842 (8.9)	656 (12.7)	<0.001
Surgery	9,401 (40.3)	7,739 (41.1)	1,662 (36.8)	<0.001
Radiotherapy	17,351 (74.3)	13,922 (73.9)	3,429 (75.9)	0.006
Chemotherapy	12,506 (53.5)	10,644 (56.5)	1,862 (41.2)	0.001
Response	6,379 (62.7)	5,108 (62.8)	1,271 (62.4)	0.766
Death	2,014 (16.4)	1,554 (16)	460 (17.9)	0.023
Oncological treatment at advanced stage				
Support care	132 (15.1)	107 (14.9)	25 (16)	0.733
Surgery	204 (27.6)	175 (28.7)	29 (22.1)	0.125
Radiotherapy	466 (63)	381 (62.6)	85 (64.9)	0.617
Chemotherapy	509 (68.8)	436 (71.6)	73 (55.7)	<0.001
Response	79 (32)	61 (30.8)	18 (36.7)	0.426
Death	85 (26.3)	66 (25.3)	19 (30.6)	0.389

*χ^2^ test.

In multivariate analysis, adjusted for stage and smoking status, older patients were found to be 15% less likely to receive surgery (OR=0.85; 95%CI 0.79–0.92), 46% had less chemotherapy (OR=0.56; 95%CI 0.52–0.61), and less than 33% had an association of two or more modalities of oncological treatment (OR=0.67; 95%CI 0.62–0.72) compared to patients under 70 years of age. Compared to younger patients, older patients were 49% more likely to receive only one modality of treatment (OR=1.49; 95%CI 1.38–1.61) and 35% more likely to receive support care (OR=1.35; 95%CI 1.21–1.51). For the radiotherapy modality, older patients were 13% more likely to receive it than young patients (OR=1.13; 95%CI 1.04–1.23) (Table 2).

**Table 2.** Multivariate analysis of the treatment modalities in patients with larynx cancer, Brazil, 2000–2017.

Modalities	Young n (%)	Older n (%)	Adjusted OR* (95%CI)
Use of at least one modality of therapy			
Yes	13,251 (48.3)	4,740 (61)	1.49 (1.38–1.61; p<0.001)
No	14,198 (51.7)	3,047 (39)	
Use of combination therapy			
Yes	14,198 (51.7)	3,047 (39)	0.67 (0.62–0.72; p<0.001)
No	13,251 (48.3)	4,740 (61)	
Surgery			
Yes	11,403 (41.5)	2,815 (36.1)	0.85 (0.79–0.92; p<0.001)
No	16,046 (58.5)	4,972 (63.9)	
Radiotherapy			
Yes	19,920 (72.6)	5,858 (75.2)	1.13 (1.04–1.23; p<italic>=</italic>0.006)
No	7,529 (27.4)	1,929 (24.8)	
Chemotherapy			
Yes	12,574 (45.8)	2,321 (29.8)	0.56 (0.52–0.61; p<0.001)
No	14,875 (54.2)	5,466 (70.2)	
Support care			
Yes	2,635 (8.8)	999 (11.4)	1.35 (1.21–1.51; p<0.001)
No	27,449 (91.2)	7,787 (88.6)	

*Adjusted by smoking and staging.

## DISCUSSION

This study evaluated 38,978 patients diagnosed with LC between 2000 and 2017, showing that patients aged ≥70 years had a higher frequency of early-stage diagnoses but were undertreated with surgery, chemotherapy, or multimodal treatments. They were more likely to receive supportive care or a single treatment modality. Radiotherapy was the most common oncological treatment.

The choice of a 70-year cutoff was based on our previous publication^3^, which found that 63.6% of LC patients were aged between 50 and 69 years, while 22.6% were 70 years or older. Epidemiological analysis also revealed differences in treatment patterns for this subgroup^3^. The use of 70 years as a cutoff is common in international studies, facilitating comparisons and discussions.

Global life expectancy rose from 66.8 years in 2000 to 73.3 years in 2019, increasing the proportion of elderly individuals. As cancer incidence rises with age, understanding treatment approaches for older patients is essential.

This retrospective analysis revealed that elderly patients (22.6% of the sample) had a median age of 75 years and clinical characteristics similar to those aged <70 years. However, the frequency of locally advanced disease at diagnosis was lower in patients over 70.

The reduced use of chemotherapy (45.8 vs. 29.8%) can be attributed to findings from meta-analysis of chemotherapy in head and neck cancer (2009), which demonstrated that while concomitant chemotherapy improves survival in head and neck cancers, its benefit diminishes with age. The 5-year survival benefit decreased from 9.8% in patients under 50 years to 3.0% in those aged 61–70 years and −0.7% in patients over 71 years, showing no significant improvement in the oldest group. Moreover, elderly patients represented only 7% of participants in the analyzed trials, limiting the understanding of intensive treatment risks and benefits. Additionally, non-cancer-related deaths increased from 15% in patients under 50 to 39% in those over 71, potentially discouraging the use of concomitant treatments in older individuals.

The limitations of this study include its retrospective design and lack of detailed data on radiotherapy and chemotherapy regimens.

In conclusion, elderly LC patients constitute a significant population subgroup. Despite similar clinical characteristics, they receive less intensive treatments, particularly surgery and chemotherapy, and are more likely to receive supportive care exclusively.
